# Barriers to nurse–patient communication in Saudi Arabia: an integrative review

**DOI:** 10.1186/s12912-019-0385-4

**Published:** 2019-12-03

**Authors:** Mukhlid Alshammari, Jed Duff, Michelle Guilhermino

**Affiliations:** School of Nursing and Midwifery, University of Newcastle, Callaghan, Australia

**Keywords:** Saudi Arabia, Nurse–patient communication, Communication barriers, Quality of nursing care, Patient satisfaction

## Abstract

**Background:**

Effective nurse–patient communication is important in improving quality of health care. However, there are several barriers to nurse–patient communication in Saudi Arabia. This is attributed to the increasing number of non-Saudi expatriate nurses providing health care to patients. In particular, there are differences in culture, religion and language among non-Saudi nurses and patients. This integrative review aims to identify and synthesize quantitative and qualitative evidence on the current practice in nurse–patient communication in Saudi Arabia and its effect on service users’ quality of care, safety and satisfaction.

**Methods:**

An integrative review based on Whittemore and Knafl’s approach (Whittemore and Knafl, J Adv Nurs 52:546–553, 2005) was used to conduct the review. Peer-reviewed articles containing any of a series of specific key terms were identified from sources such as CINAHL, EMBASE, Medline, PubMed and PsychINFO. The review included studies that focused on nurse–patient communication issues, communication barriers, and cultural and language issues. The search was limited to papers about the Saudi Arabian health system published in English and Arabic languages between 2000 and 2018. A data extraction form was developed to extract information from included articles.

**Results:**

Twenty papers were included in the review (Table 1). Ten papers employed quantitative methods, eight papers used qualitative methods and two used mixed methods. The review revealed two major themes: ‘current communication practices’ and ‘the effect of communication on patients’. Some of the communication practices rely on non-verbal methods due to a lack of a common language, which often results in the meaning of the communication being misinterpreted. Many non-Saudi nurses have limited knowledge about Saudi culture and experience difficulty in understanding, and in some cases respecting, the cultural and religious practices of patients. Further, limited nurse–patient communication impacts negatively on the nurse–patient relationship, which can affect patient safety and lead to poor patient satisfaction.

**Conclusions:**

Current nurse–patient communication practices do not meet the needs of Saudi patients due to cultural, religious and language differences between nurses and patients. The barriers to effective nurse–patient communication adversely effects patient safety and patient satisfaction. Further research from the perspective of the patient and family is needed.

## Background

The concept of communication is a complex process of exchanging information, thoughts and feelings between individuals using a common system of signs, symbols or behaviors. This process consists of several components, including sender, receiver, context, medium, message and feedback. For communication to occur, a message (information, thoughts and feelings) is transmitted by the sender (also called the encoder) through a suitable medium in a given context to a receiver (also called the decoder), who then provides feedback [1].

In the health-care setting, several theoretical and conceptual approaches have been employed to improve health outcomes, including patient-centered communication [2–6]. Patient-centered communication has been identified as an essential component in delivering quality health services [4]. High-quality patient-centered communication has been shown to help patients strengthen their relationship with nurses, handle their emotions, understand important information regarding their illness, deal with uncertainty, and participate more fully in decisions regarding their health [2, 4].

Nurse–patient communication plays an important role in improving not only patient’s relationship with the nurse, but also the patient’s own perception of the treatment process and outcome. Moreover, having effective communication skills is essential for health-care providers’ practice and their ability to understand the clinical symptoms and psychological and emotional needs of their patients. Patient-centered communication enables the building of therapeutic relationships, which helps health-care providers apply intelligent, sensitive and collaborative approaches to communicate with patients about their services [2, 5, 7–10].

Despite the potentially significant benefits of patient-centered communication, there have been communication barriers identified across a number of different practice settings worldwide [11–14]. For instance, limited knowledge and understanding of the culture and language of a health system on the part of a patient has been shown to limit the communication process between patient and clinician [13–16]. These barriers are influenced by several factors including cultural and language diversity [12]. These communication barriers can affect health outcomes, quality of health care, patient safety and patient satisfaction.

Nurse-patient communication is a challenge in the Saudi Arabia health system because many of the nurses are expatriates and don’t speak Arabic. This issue is not unique to Saudi Arabia, due to increasing levels of immigration into developed countries such as United States of America and Australia, there is increasing cultural and linguistic diversity between nurses and their patients [12, 14]. In fact, a recent systematic review [13] suggested that such communication barriers are common to many countries and they adversely affect the overall quality of health services.

There has been growing interest in research on nurse–patient communication in Saudi Arabia, including quantitative and qualitative studies [17–21], but despite the growing evidence base no study has focused on the communication experiences of patients. Furthermore, no study has examined if the patient’s communication experience impacts satisfaction with their nursing care.

## Aim

This integrative review aims to identify and synthesize quantitative and qualitative evidence on the communication practices among nurses and patients in Saudi Arabia and their effect on patient satisfaction, quality of care and safety.

## Methods

An integrative review was chosen to merge diverse methods, and synthesize findings from both qualitative and quantitative studies [22]. The integrative review used Knafl and Whittemore’s methodology [23]. This approach involved a five-stage process: (1) identify the purpose of the review, (2) search the relevant literature, (3) evaluate and extract data, (4) analyze or synthesize the data, and (5) present findings [23]. The quality of the included papers in this review were evaluated using the Mixed Methods Appraisal Tool (MMAT) [24].

### Inclusion criteria and search strategy

The review included papers written in Arabic and English and focused on Saudi Arabian healthcare. Studies were included if they focused on nurse–patient communication, including communication barriers such as language and cultural issues and their effects on patients.

CINAHL, EMBASE, PubMed, Medline and PsychINFO databases as well as Google Scholar were searched for articles published between 2000 and 2018. These databases and the time limit were chosen to ensure a comprehensive search and a sufficient breadth and depth in the retrieved literature. A two-stage search approach was utilized to facilitate the search process. An initial search was conducted in Medline and EMBASE. Subsequently, the identified key words and search items were modified and used to search across CINAHL, PUBMED and PsychINFO. The search items that facilitated the search process were barrier* or facilitat* or limit* or challeng* or difficult* or obstacle* or problem or issue AND communicat* or language or cultur* AND nurs* AND Saudi* AND quality or satisf* or effectiv* or impact.

### Data extraction and synthesis

**Fig. 1:**
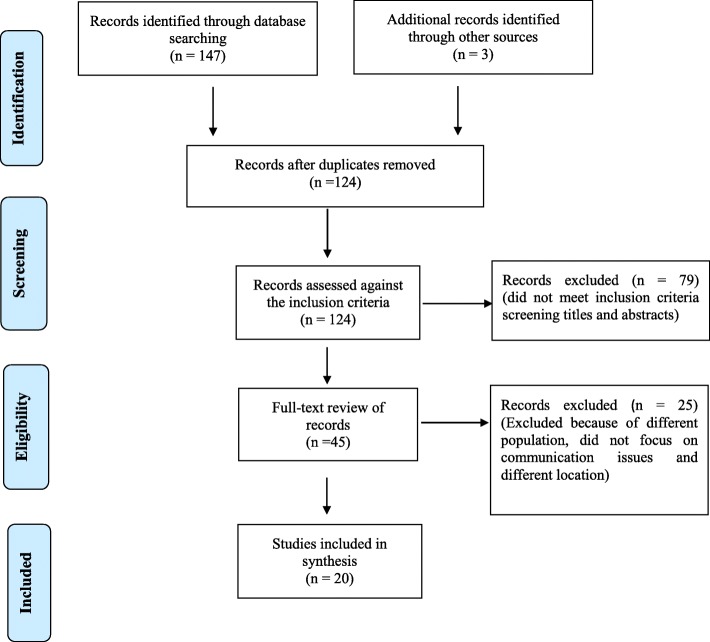
PRISMA chart of article selection

The integrative review contained several stages in the selection of papers. The Preferred Reporting Items for Systematic Reviews and Meta-Analyses (PRISMA) flow chart (Fig. 1) represents the process. First, the titles and abstracts of all the papers identified from the scientific databases were screened against the inclusion criteria. Second, the abstracts of all the included titles were reviewed to identify those that required full text review. The final stage of the selection process involved screening the full text articles to agree on those to include in the final synthesis. Two reviewers independently managed the selection process.

A pre-defined data extraction form was developed to guide the process of data extraction. The tool was developed and structured according to the systematic review reporting guidelines [25]. The data extraction form was structured into sub-sections, which included description of study (author, year of publication and title of paper), methods (study design, methods, sampling and sample size) and findings.

### Critical appraisal

The quality of the papers was assessed using the Mixed Methods Appraisal Tool (MMAT). This tool has been validated and widely used to asses quality of papers with different methods [24]. MMAT has three categories of quality score, including low (a score below 25%), medium (a score of 50%) and high (a score of 75% and above). All of the included studies were checked on the basis of data relevance as well as methodological rigor.

## Results

### Study characteristics

A total of 150 records were reviewed from CINAHL, EMBASE, PubMed, Medline, PsychINFO and Google Scholar, of which 26 duplicates were deleted. From this, 124 titles and abstracts were assessed against the inclusion criteria, with 79 excluded. A full text review of the remaining 45 documents was undertaken. Twenty five manuscripts were later excluded, 10 because of the population, seven were excluded because it did not focus on nurse-patient communication issues and its effect on health outcomes; and eight were excluded because the study setting did not include Saudi Arabia healthcare context. Overall, data was extracted from 20 full text articles and included in the final synthesis (Fig. 1). Of the 20 papers included, eight used qualitative methods, 10 used quantitative methods and two used mixed methods (Table 1). Further, 15 of the included studies targeted nurses’ perspectives of the communication and five focused on patients’ perspectives of communication. A quality assessment of the included papers found that most (12 out of 20) were asssessed as high quality, while the remaining papers (eight out of 20) were assessed as medium quality.

**Table 1:** Characteristics of the included studies

Author/year	Study design	Methodology	Participants	Settings	Quality of the paper	Key findings related to review
MD Al-Mendalawi [26]	Cross-sectional survey	Quantitative	116 patients	In-patient at tertiary referred hospital	Medium	Patients are satisfied with health services regardless of language barrier.
ZA Mani and MA Ibrahim [20]	Cross-sectional survey	Quantitative	77 nurses	ICU at tertiary referred hospital	High	There are communication difficulties between nurses and patients in end-of-life care.
A Shubayra [27]	Descriptive Semi-structured, face-to-face interviews	Qualitative	9 nurses	Peritoneal dialysis at tertiary referred hospital	High	Language barriers impeded effective nursing education to patients.
M Silbermann, RM Fink, S-J Min, MP Mancuso, J Brant, R Hajjar, N Al-Alfi, L Baider, I Turker and K ElShamy [21]	Descriptive survey	Quantitative	776 health-care providers	Oncology department at three tertiary referred hospitals	High	There are numerous communication difficulties in palliative care.
WA Suliman, E Welmann, T Omer and L Thomas [28]	Descriptive survey	Quantitative	393 patients	Three national guard health affairs facilities	High	There are communication barriers that influence nurse–patient relationships.
G Abudari, H Hazeim and G Ginete [29]	Phenomenological design	Qualitative	10 nurses	Oncology department at tertiary referred hospital	High	Non-Muslim nurses are facing several challenges in taking care of Muslim cancer patients.
AH Al-Doghaither [30]	Not reported	Quantitative	450 patients	In-patient at university hospital	High	Different levels of satisfaction are perceived by patients related to nurses competency level or interpersonal skills
H Aljadhey, MA Mahmoud, MA Hassali, A Alrasheedy, A Alahmad, F Saleem, A Sheikh, M Murray and DW Bates [31]	Exploratory design	Qualitative	65 health-care providers	Secondary level and Private hospital	Medium	Communication barriers threat patient safety (medication error).
AF Almutairi, G Gardner and A McCarthy [32]	Cross-sectional survey Case study design	Mixed method	319 nurses	In-patient at tertiary referred hospital	High	Nurses from different cultures have different perceptions about the safety environment.
BM Hammoudi, S Ismaile and O Abu Yahya [33]	Cross-sectional survey	Quantitative	367 nurses	In-patient at four tertiary referred hospitals	Medium	Nurses’ languages and cultural diversity influence medication administration as well as reporting errors.
A Khalaf, A Westergren, Ö Ekblom, HM Al-Hazzaa and V Berggren [34]	Explorative design	Qualitative	15 nurses	In-patient at secondary health level hospital	High	There are differences in language, religion and culture among nurses providing health services.
AG Mohamed [35]	Cross-sectional survey	Quantitative	343 nurses	Five hospitals at different health levels	Medium	According to nurses, patients can be dissatisfied due to many reasons including communication.
J Mebrouk [36]	Phenomenological design	Qualitative	5 nurses	In-patient at tertiary referred hospital	High	Saudi nurses have enough knowledge regarding language, religion and cultural whereas expatriate lack knowledge. Expatriate nurses usually use non-verbal communication.
H Alabdulaziz, C Moss and B Copnell [17]	Explanatory sequential design	Mixed methods	234 nurses	Paediatrics at secondary health level hospitals	High	There are differences in language, religion and culture among nurses and patients.
DN Alosaimi and MM Ahmad [18]	Descriptive Semi-structured interviews	Qualitative	20 nurses	In-patient at tertiary referred hospital	High	Limited verbal communication and limited knowledge of religion and culture exist among expatriate nurses.
MA Atallah, AM Hamdan-Mansour, MM Al-Sayed and AE Aboshaiqah [37]	Cross-sectional design	Quantitative	100 patients	In-patient at tertiary referred hospital	Medium	Different levels of patient satisfaction occur depending on either nurses’ competency or interpersonal skills.
E Sidumo, VJ Ehlers and S Hattingh [38]	Descriptive, exploratory study design	Quantitative	50 nurses	Obstetric unit at secondary level hospital	Medium	Limited knowledge about cultural and religious practices exists among expatriate nurses.
H Al Fozan [39]	Cross-sectional design	Quantitative	302 patients and family caregivers	In-patient of national guard health affairs facility	Medium	Patients are satisfied with Saudi nurses who have same language, culture and religion.
P Halligan [19]	Phenomenological design	Qualitative	6 nurses	ICU at tertiary referred hospital	Medium	Patients misinterpret some of the expatriate nurses’ non-verbal communication.
M Van Bommel [40]	Phenomenological descriptive	Qualitative	63 nurses	CCU at tertiary referred hospital	High	There are language, cultural and religious diversity among expatriate nurses and patients in ICU.

### Identified themes

The major themes were grouped into two themes: ‘current communication practices’ and ‘the effect of communication on patients’ (Table 2). The sub-themes identified from the ‘current communication practices’ theme were language, religion and cultural diversity; communication practices; and communication barriers. The sub-themes identified from the ‘effect of communication on patients’ theme were quality of care and patient satisfaction.

**Table 2:** Emerging themes and sub-themes

Theme	Sub-theme	Number of papers
Current communication practices	Language, religion and cultural diversity	7
	Communication practices	4
	Communication barriers	6
Effect of communication on patients	Quality of care and patient safety	5
	Patient satisfaction	5

## Current communication practices

In Saudi Arabia, the nursing workforce across almost all health facilities is dominated by non-Saudi nurses, primarily from the Philippines and India, supplemented by nurses from the USA, UK, Australia and various European countries [40]. The increasing number of non-Saudi or expatriate nurses has created several challenges in the delivery of health care. In particular, the challenges are ascribed to several factors, mostly linked to cultural, language and religion differences. The current communication practices present a barrier to patient-centered interaction between nurses and patients [19, 29]. These challenges are described below.

### Language, religion and cultural diversity

Seven of the included papers explained that there are differences in language, religion and culture among nurses providing health services to patients in Saudi Arabia [17–20, 34, 38, 40] with the language, religion and culture of non-Saudi nurses differing from their Saudi patients. Unlike the expatriate nurses, almost all patients in Saudi Arabia speak Arabic and share the same cultural values, norms and religion [40].

Khalaf, A et al. (2014) stated that the religious-cultural norms and values of Saudi patients appear as entirely new to many non-Saudi nurses [34]. Some cultural or religious practices, such as gender segregation or females covering their hair or faces, do not appear to be rational to many nurses. In addition, some religious-cultural practices, which lead female patients to have a preference for female practitioners when seeking health care, are sometimes seen as irrational by non-Saudi nurses.

Consequently, multiple studies have found that non-Saudi nurses experience difficulty in understanding, and in some cases respecting, the cultural and religious practices of patients [20, 38]. This was attributed to the fact that most of these expatriate nurses have limited knowledge about the practices of their patients. In particular, the nurses have limited knowledge about the cultural and religious practices of patients [18, 38]. For instance, a previous study showed that more than half of all expatriate nurses lack knowledge of Saudi culture [38]. Practically, this makes it difficult for the nurses to understand the communication dynamics of patients in the process of seeking health care.

The differences in language, culture and religion are significant factors that can directly influence the communication experience of the patient. These factors cannot be overlooked in the delivery of health care [17, 19, 38]. For example, cultural and traditional practices including the use of herbal medicine, preference for breastfeeding and practice of burying the placenta are believed to improve health and prevent illness of both baby and mother. Along with strong family bonds, these factors have historically played a significant role in delivery of Saudi Arabian health care. In addition, religious beliefs and practices such as fasting and praying are perceived as relevant factors in the recovery of patients [36, 40]; however, some non-Saudi nurses have demonstrated difficulty in understanding these religious-cultural practices that contribute significantly in the delivery of health care.

Nor is this a new phenomenon. Differences in language between nurses and patients have historically created problems in the health systems of Saudi Arabia, particularly in some routines of nurses. Two studies identified that language diversity causes significant challenges in caring for patients with life-threatening conditions; in particular, when discussing patient wishes in terms of their care and during sessions of health education [20, 29]. Other studies identified that nurses experienced difficulty in understanding patients, particularly when seeking information during health-care delivery, such as taking the history of the patient [18, 19]. Several studies have suggested that the phenomenon not only poses significant challenge to the nurses but also to the patients and their families [17, 20, 34]. In particular, some nurses avoid conversations with patients or their families due to language differences.

### Communication barriers

Six studies have shown that there are numerous interpersonal therapeutic communication barriers existing between nurses and patients as well as family caregivers [17–21, 27]. These barriers occur at different levels, including nurses to patients as well as at the inter-professional level [21, 34]. The inter-professional communication barriers are the barriers occurring at the health provider level, mostly between nurses from Saudi Arabia and non-Saudi expatriate nurses. The communication barriers occurring at different levels of the health system are ascribed to several factors, again mostly linked the differences in language, culture and religion [17, 18, 20, 26, 29, 34].

Consequently, the limited Arabic language, culture and religion knowledge have negatively impacted on the delivery of health care, particularly by limiting the communication processes between non-Saudi expatriate nurses and patients [17, 18, 20]. In particular, the language difficulties limit nurses’ ability to effectively communicate with patients [17, 18, 20]. Similarly, the communication barriers occurring at inter-professional level affect health services planning [31].

As well as the in-service training programs noted above, the health authorities have employed several strategies to overcome these challenges, particularly at the health facility level. As described by Almutairi, AF et al. (2013), these strategies include the use of interpreters or family member to translate conversations [32]. Although these strategies can be useful, they are not always as effective as needed. For example, both translators and family members have been shown to deliver an incomplete or unclear rendering of the conversation to the patient, which could adversely influence health-care practice [32].

It has been argued that the provision of interpreters in all hospitals in Saudi Arabia would be a good step towards improving communication [32]. However, many of these translators would need training in personal and professional interpretive skills, particularly in the area of medication administration [41]. Although most current interpreters are competent in the English language, their skills are limited to the understanding of medical terms and jargon.

### Communication practices

Four studies identified communication practices as a key theme. They identified several communication practices currently employed by nurses in health facilities across Saudi Arabia that are perceived to be effective in interpersonal therapeutic communication [18, 19, 29, 36]. Existing evidence suggest that most nurses employ non-verbal communication practices in their communication with patients. Predominantly, this takes the form of gestures and signs, and sometimes therapeutic touch as well as smiling [29, 36]. In some instances, these non-verbal communication practices help patients understand the process of health-care delivery. In particular, recent evidence suggests that the non-verbal communication appears to reassure patients and their families about the medication processes, which provides them with a degree of relief [29].

However, despite the increasing use of non-verbal communication practices, two studies have highlighted that such communication is frequently misinterpreted by patients [19, 36]. Two examples are the clicking of fingers to attract the patient’s attention, and the use of direct eye contact with patients. To Saudi patients, the clicking of fingers to attract attention can be understood as offensive [19], while direct eye contact by female nurses to male patients could be understood as a rude behavior [36].

In addition to the non-verbal communication, some nurses communicate verbally to facilitate patient-centered interaction [18]. The verbal interactions are usually limited and delivered through few Arabic/Islamic terms. A recent study suggested that words such as “Bismillah” or “Alhamdillah” – which translate as “in the name of Allah” and mean to start with the blessing of God – are mostly used prior, during and after medication processes, largely to make the patient feel more comfortable [18].

The Ministry of Health in Saudi Arabia has responded to these issues by instituting cultural training and orientation programs for nurses. This training is delivered as an in-service program with the primary aim of exposing nurses to religious-cultural practices in the delivery of health care in Saudi Arabia [40]. However, despite this development, two studies have suggested that these courses appear to have limited impact and lack the ability to meet the needs of such nurses [32, 40]. In particular, this training and orientation program has a short duration and scope, and it has been noted that in order to address such communication gaps, improvements would be required in the content, structure, duration and intensity of the program [32].

## Effect of communication on patients

### Quality of care and patient safety

Five studies suggested that a comprehensive understanding of the culture, religion and language of a geographical setting play significant role in improving the quality of care and safety of the population [27, 28, 31–33]. In particular, nurses who have some knowledge and understanding of Saudi religious-cultural practices are perceived to be more competent in delivering care compared to those with limited competency [31]. For instance, some expatriate nurses continue to struggle with communication and subsequently feel frustrated, particularly in understanding aspects of the patient’s culture and religion [19]. One study proposes that a deep understanding of some elements of the religious-cultural attributes – such as Muslim lifestyle, hygienic practices, ways of dressing, and gender segregation by nurses – is necessary to improve the quality of care and safety of patients [40].

Four studies highlighted that communication barriers caused by the differences in religious-cultural practices have implications for the safety of the patients [27, 31–33]. These challenges are experienced in areas such as medication safety and the emotional, psychological, physical and spiritual domains of patients and family members. In some instances, the challenges affect not only patients but also the nurses delivering care to patients.

Three studies suggested that where nurses have religious-cultural practices that differ from those of their patients, safety can be impacted [27, 32]. For example, patients may find it difficult to adhere to the nurse’s instructions, resulting in a clear threat to patient safety [26, 31, 33]. Another threat to patient safety is caused by miscommunication between nurse and patient or between health professionals. Aljadhey, H et al. (2014) have suggested that language barriers could account for an increase in medication errors [31]. Similarly, Hammoudi, BM et al. (2017) found that some non-Saudi nurses are hesitant to report medication errors or subsequent adverse effects on the patient for fear of disciplinary action [33].

### Patient satisfaction

Five studies highlighted that patients exhibit different levels of perceived satisfaction from different aspects of health care in Saudi Arabia [26, 30, 35, 37, 39]. Approximately 75% of patients reported being satisfied with the health care they have previously received; however, while these patients are generally satisfied with the technical competence of nurses [30, 37], approximately half of them are dissatisfied with the interpersonal therapeutic communication of most expatriate nurses.

In particular, nurses who share the same language, culture and religion as the patient are perceived to communicate professionally and clearly, to respect culture and religion, and to maintain patient’s privacy. This contributes to building a good relationship between nurse and patient, resulting in an improved satisfaction with patient care [36, 37, 39]. Although patients recognize that expatriate nurses are generally technically competent, patients are less satisfied with interpersonal therapeutic communication, as they perceive nurses to be ignorant of their language, culture and religion [37]. This can be perceived as disrespect, which might contribute to reported levels of violence towards nurses. A previous study highlighted that communication barriers was one of the important factors in work-related violence [35].

## Discussion

This review aimed to synthesize evidence on nurse–patient communication practices among nurses and patients in Saudi Arabia and their effect on patients’ quality of care, safety and satisfaction. The review included 20 papers in the final synthesis. The review findings suggest that there is a diversity in the language, religion and culture of nurses providing health-care services in Saudi Arabia. In particular, nurses providing health care in Saudi Arabia are largely expatriate and tend to have limited knowledge about Saudi language, religion and culture. Consequently, expatriate nurses rely mostly on non-verbal communication strategies to interact with patients. The review findings suggest that the cultural and language training provided to expatriate nurses is not fit for purpose. Bozionelos [42] qualitative study of 206 expatriate nurses in Saudi Arabia found that nurses are provided with limited face-to-face training due to an overall pressure on nursing services related to the nursing shortage in Saudi Arabia. The limited knowledge about the language, religion and culture of non-Saudi nurses, together with the current communication practices described earlier, have significantly contributed to nurse–patient communication barriers in Saudi Arabia. The review findings suggest that the interpersonal therapeutic communication barriers occur among health care professionals as well as between nurses and patients. In addition, the review findings highlighted that the communication barriers have significant influence on the outcomes of health-care service delivery; in particular, communication barriers have negative effect on the perceived quality of care, patient safety and patient satisfaction.

It is noteworthy that no study has addressed nurse–patient communication experiences from patient perspectives in Saudi Arabia. Given the nurse–patient communication challenges discussed in this paper, future patients with complex needs may face specific challenges in accessing health-care services due to the nature of their conditions, which requires frequent attention from nurses. In particular, patients may require attention in therapeutic communication to make complex and significant medical decisions. In addition, some patients may need more attention from nurses in the process of treatment. Consequently, to improve access to treatment for patients, it is important not only to understand the burden of their conditions but also to address the therapeutic communication issues with service providers, particularly nurses.

Based on these review findings and Bozionelos [42] study, some of the recommendations for clinical and policy practices should include the provision of adequate cultural and language training before expatriate nurses leaving their home country; and the implementation of mentorship programs to support and guide expatriate nurses [42]. This can help to improve the communication between nurses and patients in the delivery of health care services. Second, the current in-service training curriculum for nurses in Saudi Arabia should be expanded, and incorporate a component on language, cultural and religious practices. This ought to carry through at least the first year of employment for all nurses, include a formal evaluation component, and be reviewed regularly by the Saudi Ministry of Health for quality and effectiveness.

In addition, the review findings recommend the following in future research. First, because current studies on nurse–patient communication issues largely use the perspective of nurses, with limited studies focusing on patients, particularly those with complex needs, future research should aim at investigating the perspectives of patient and family members on nurse-patient communication issues. Second, researchers and clinicians should aim to use a mixed methods approach to examine the perspectives of both nurses and patients on communication issues. This can help achieve convergence in data analysis. Finally, future research should aim at designing interventional studies to examine the effectiveness of the nurse-patient communication strategies on patient satisfaction and health outcomes.

## Strength and limitations

The strengths of this study include the use of a systematic approach to search data from relevant scientific databases, revealing most available papers on the subject of nurse–patient communication and associated issues such as cultural, religious and language challenges, perceived quality of care, patient safety and patient satisfaction.

Second, the review used a data extraction form to extract all relevant information that met the inclusion criteria. The data extraction form was developed using relevant methodological standards and criteria. Similarly, the review was not limited to any particular sphere of health care, and so could identify all relevant papers, irrespective of any particular health condition being examined.

The study also has several limitations. First, the study was necessarily limited to a selection of specific search items, and so could have missed some relevant papers. Further, the study was limited to papers focusing on Saudi Arabian health care, and so cannot be generalized to other settings. However, the use of a systematic approach [23] – including data searching, data extraction and collaboration with experts in the field – attempted to reduce the impact of the limitations.

## Conclusion

The study concludes that language, culture and religion differences exist between patients and nurses in Saudi Arabia, primarily due to the preponderance of expatriate nurses in working in the Saudi Arabian health-care system. These differences create barriers to clear and effective communication and produce a negative impact on health outcomes for patients in Saudi Arabia. Moreover, the findings of this review indicate a need to improve communication between patients and health-care providers in order to provide safety and high-quality practice in Saudi Arabia, which will contribute to higher quality of care and patient satisfaction. There should be a focus on research in extensive training programs for nurses.

## Data Availability

All data generated or analyzed during this study are included in this published article (and its supplementary information files).

## Abbreviations

MMAT: Mixed Methods Appraisal Tool
PRISMA: Preferred Reporting Items for Systematic Review and Meta-analysis
